# Streamlined Postprocessing of NMR Structures with the Molecular Restrainer: A Universal Tool for High-Quality Protein–Ligand Models and Non-Standard Amino Acid Residues

**DOI:** 10.3390/ijms26115091

**Published:** 2025-05-26

**Authors:** Jiří Mareš, Guneet Singh Tarang, Dmitriy Marin, Mehdi Mobli, Stephane Redon, Julien Orts

**Affiliations:** 1 Department of Pharmaceutical Sciences, University of Vienna, Josef-Holaubek-Platz 2, 1090 Vienna, Austria; jiri.mares@univie.ac.at (J.M.); guneet.singh.tarang@univie.ac.at (G.S.T.); 2 OneAngstrom, 38000 Grenoble, France; dmitriy.marin@oneangstrom.com (D.M.); stephane.redon@oneangstrom.com (S.R.); 3 Australian Institute for Bioengineering and Nanotechnology, University of Queensland, St Lucia, QLD 4072, Australia; m.mobli@uq.edu.au

**Keywords:** molecular restraint, NMR spectroscopy, 3D-structure refinement

## Abstract

We present a molecular restrainer tool capable of energy-minimizing NMR-derived structures using the universal force field (UFF) and NOE-derived distance restraints. The implementation is a part of SAMSON (version 2024 and newer), an integrative molecular design platform. The strength of our tool lies in its ability to swiftly process any molecule and molecular complex without the need to create force field parameters and topology files. We show, using examples, that the quality of these refined structures is significantly improved compared to the starting structures derived by CYANA. Currently, the implementation is targeted toward the postprocessing of structures derived by the software CYANA but can easily be adapted to other molecular restraint formats. This tool enables the generation of publication-ready protein–ligand complex structures for PDB deposition, requiring minimal additional effort beyond the initial NMR structure calculation.

## 1. Introduction

The distance restraints obtained by the NOE effect have been the major source of structural information derived from NMR spectroscopy. Its theory has been established for many decades, with the first protein structure solved in the mid-eighties [1], followed by advanced studies, including the full relaxation matrix treatment, shortly after, in 1986 [2].

The lack of available theoretical understanding is, therefore, not the bottleneck for the spectroscopist. Also, computational tools to derive the structures of biomolecules have been mostly matured in the early-to-mid nineties (e.g., ref. [3]). For a protein composed of standard amino acids, the structure calculation is currently well-automated in several available tools due to the availability of force fields and programs to handle them, such as Xplor-NIH [4], CYANA [5], ARIA [6] and its web interface [7], UNIO [8], and ASDP [9], or in more molecular dynamics-oriented tools, including GROMOS [10] and GROMACS [11].

The situation is significantly more difficult for chemical moieties other than those forming the repeating units of standard biopolymers, such as cofactors or drug molecules. In biomolecular complexes, arguably the most important systems, especially in drug discovery, setting up a calculation involving complexes imposes a challenge beyond the standard toolbox available to the NMR spectroscopist.

For structure calculation with CYANA, the development of parameter files has been automated since 2015 [12]. The torsion-angle dynamics, together with a minimalistic set of force-field atom types, enables efficient structure calculation. It is desirable to refine the resulting structure in a force field with other degrees of freedom, including bond lengths and bond angles, as well as common non-bonding parameters, including both van der Waals and electrostatic terms. To obtain parameters for the ligand, automatic tools and servers exist: Antechamber [13] and its web interface [14], SwissParam [15], CharmGUI, [16] and ATB server [17], with which parameters in different formats, compatible with different force field parameters, can be obtained. These are, nevertheless, intended for molecular-modeling projects, which commonly require a significant time investment. On the other hand, to our knowledge, no tool is available for structure refinement that does not require specific knowledge about molecular modeling and ensures a seamless process. Therefore, we present such a tool, implemented within a modern molecular design software, SAMSON [18], a quickly growing platform for integrative molecular design. The fully flexible molecular model uses the universal force field (UFF) [19], with a small number of force-field atom types, spanning nearly the whole periodic table. As all parameters are only related to atom types, while all the bonding and nonbonding parameters are derived using combination rules, the force field can handle any molecule. The first version of the tool, which we call molecular restrainer, uses distance restraints in the CYANA format and the UFF to minimize/refine a supplied set of NMR-derived structures in a few simple and intuitive steps. To verify the employed force field’s suitability, we test here several protein systems with small organic molecules, as well as peptides with non-standard amino acid residues. We show that the molecular restrainer is capable of improving NMR-derived structures or maintaining the accuracy of the supplied structures. Furthermore, it is able to correctly define the N-terminal and C-terminal groups of proteins to make them chemically correct.

## 2. Results

### 2.1. Characterization of the Data

We selected PDB protein structures from the www.rcsb.org (accessed 21 May 2025) database obtained by solution NMR, calculated by CYANA, and containing one or more “non-polymer entries”. Further, the restraint files compatible with CYANA were required for selection. Disregarded were those with possible biasing features (such as all carboxyl groups in the form of COOH). The PDB entries chosen are 2N3Y [20], 2NBN [21], 5LQV [22], 6R1V [23], and 7NM2 [24]. These represent proteins with ligands. Table 1 shows the ligands or non-standard residues present in these PDB structures.

**Figure 1:**
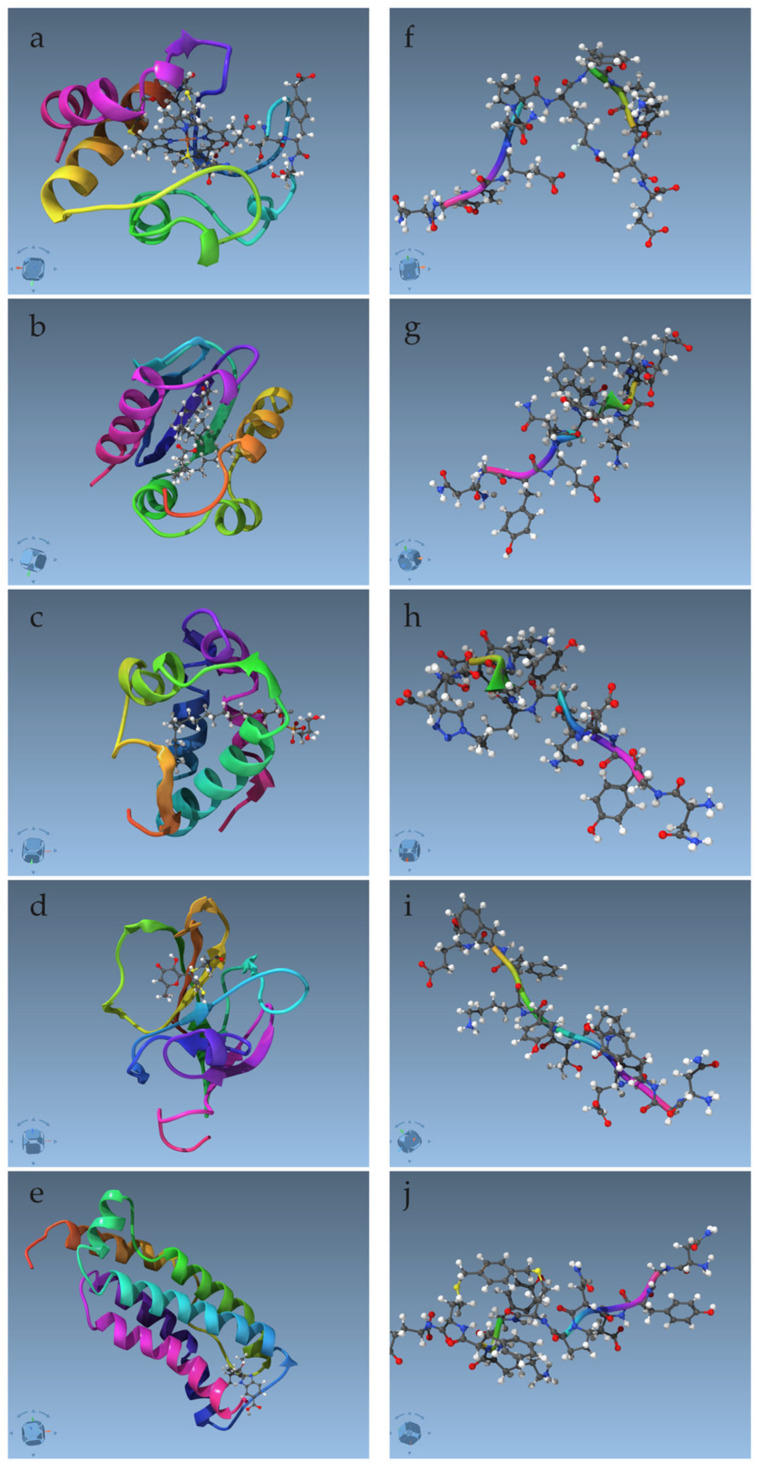
Molecular systems tested in this study, with proteins and complexes on the **left** (**a**–**e**) and peptides on the **right** (**f**–**j**). Graphics in the default visualization scheme of SAMSON. Structures minimized by SAMSON are displayed. The protein–ligand complexes are in the same order as in Table 1; the peptides are in the same order as the second half of Table 1.

The five selected protein structures contain a variety of organic molecules, in addition to the standard amino-acid residues. 2N3Y is a structure of a cytochrome composed of 104 amino acid residues, with a covalently bonded iron-containing heme cofactor and an amino acid derivative (see Figure 1a and Table 1). The NMR restraints file (.mr) contains, overall, 6876 distance restraints, of which 582 include the heme group. The structures from CYANA are refined by the molecular dynamics in AMBER [25], using parameter files obtained from a different study [26]. 2NBN is a structure of a sterol carrier protein of 111 residues, solved solely with CYANA, like all of the following examples, with two molecules of palmitic acid. The restraints file contains 1907 distance restraints, of which 96 include the palmitic acids. 5LQV is a structure of a lipid-transfer protein 2, in complex with lysophosphatidylglycerol. The restraints file contains 1779 distance restraints, of which 56 include the lipid molecule. 6R1V is a structure of sortase A, composed of 148 residues, and covalently linked to a compound containing two heterocycles. The restraints file contains 1490 distance restraints, of which 10 include the heterocyclic compound. 7NM2 is a structure of a mixed lineage kinase domain with 157 residues, with a non-covalently bound ligand containing two cyclic structures. The restraints file contains 1979 distance restraints, of which 58 include the ligand. All these datasets are available at www.rcsb.org (accessed 21 May 2025).

In addition to these, we collected a set of five peptides with non-standard amino acid residues and bonds and non-canonical side-chain linkages [27]. These peptides, targeting the phosphotyrosine-binding domain of Mint2, have been studied in connection with Alzheimer’s disease [28]. These dodecapeptides have 138–233 distance restraints for the cyclized peptides and 74 for the linear WT-4, the structure “i” of Figure 1.

**Figure 2:**
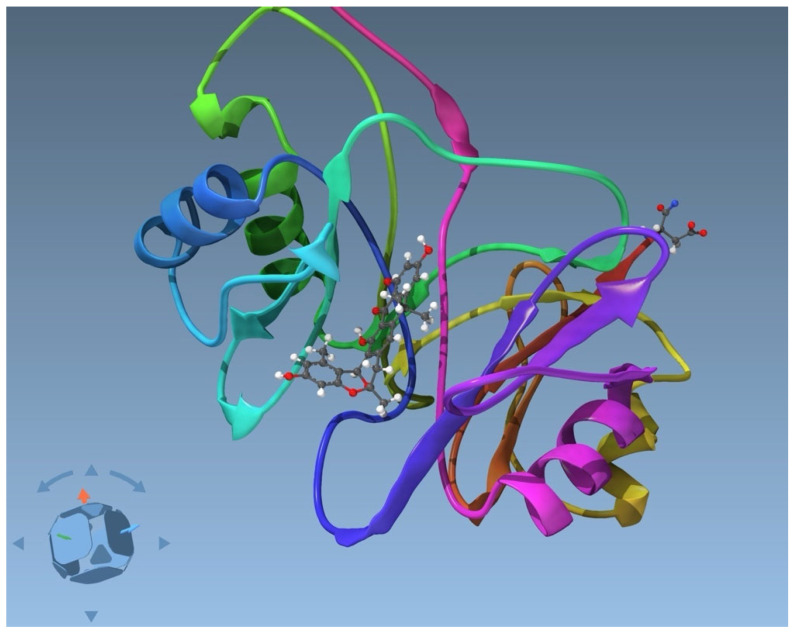
Protein–ligand complex abx-ABX, a glyoxylase-like protein with an anthracene-derivative ligand.

As a last system, abx-ABX is a glyoxylase-like protein with 245 amino acid residues (Figure 2) [29], catalyzing an oxa-Diels–Alder reaction. For this complex solved by NMR, there are 3996 distance restraints, of which 48 include the ligand. For this protein, we have all the information from the structural calculation in CYANA at our disposal, such as a consistent list of atom names and the molecular restraints for the small molecule.

### 2.2. Performance of the Refinement

#### 2.2.1. Proteins and Complexes

In this study, for the protein–ligand set downloaded from the PDB database, the restraints, including the ligand or non-standard residues, cannot be used due to the inconsistency between the names of the ligand molecule and its atom names compared to the names in the deposited .pdb files. The organic compounds are nevertheless present in all the systems of this study. In SAMSON, these protein structures are then minimized using only restraints between the atoms of the standard residues, whereas the set of peptides is minimized also using the restraints, including non-standard residues. We used the wwpdb.org validation tool to obtain concise measures and evaluated the quality of the original deposited structures before and after minimization. The basic scores, calculated by MolProbity [30], include clashscore as the number of close contacts per thousand atoms, Ramachandran outliers as a percentage of outliers with respect to the number of residues, and side-chain outliers, similar to the torsion-angle outliers from Ramachandran plots but for amino acid side chains. The results are collected in Table 2. From the clashscore, Ramachandran, and side-chain percentile scores, it can be seen that the minimized structures are improved in the clashscore and the side chain conformations. The Ramachandran scores, which are already very good in the deposited structures, do not change significantly. An exception in this set of structures is 2N3Y, which has been refined by molecular dynamics in AMBER. In this case, minimization in SAMSON did not clearly improve the structures.

For the abx-ABX complex, there is no inconsistency between the atom names in the pdb and restraints file, and thus, 29 out of 48 protein–ligand restraints could be used. The remaining restraints contain pseudoatoms from the ligands, for which the names introduced by the cylib of CYANA are not compatible with SAMSON. Also in this case, the minimization in SAMSON improves the clashscore and side-chain score, whereas the Ramachandran statistics remain very good but slightly worse.

As a second measure, we use the target function value calculated by CYANA. Only the restraints between atoms of the standard residues are used. As seen from Table 2, minimization with the molecular restrainer in SAMSON significantly improves the target function in all cases.

#### 2.2.2. Peptides with Non-Standard Residues

The second set of structures, peptides with non-standard residues and non-standard linkages, was significantly different from the previous one. In this case, we had access to the CYANA library for all the non-standard residues. The library is constructed with all atoms explicitly included, and the relatively low number of distance restraints results in nearly flawless structures already from the CYANA calculations. Minimization using SAMSON starts from geometries that are already nearly ideal with respect to both UFF parameters and applied restraints. According to the wwpdb.org validation tool, there are also only slight changes. From the five peptides, the seemingly non-ideal scores are caused by the presence of non-standard residues. In the case of the minimized structure APP-Lac-7, the validation server interprets the bonded atoms as involved in a non-bonded steric clash. In another case, the side-chain rotamer scores remain non-ideal in the immediate neighborhood of the non-standard residues or non-standard side-chain linkages. There is, therefore, no evidence of performance problems with the molecular restrainer.

## 3. Discussion

### 3.1. Justification of the Use of UFF

The use of UFF proved to be fruitful, despite the seeming simplicity of the UFF parameters. Actually, the UFF has been carefully parametrized with additional parameters, uncommon to the biomolecular force fields. For example, the force constants of bonds, angles, and torsions depend on scaled nuclear charges for the different atom types. Furthermore, the interatomic bonding force is parametrized both for the common harmonic potential, as well as for the Morse potential. From the specific non-bonding parameters, the partial charges, commonly tailored using charge-density fitting [13], employ a well-established approach of charge equilibration [31]. These are, however, currently not implemented in SAMSON, as described in ref. [32], which is consistent with the implementation of UFF in OpenBabel [33]. This simplification may be seen as a shortcoming, but it also has an advantage in avoiding the need for a solvation model to moderate the strong electrostatic interactions.

#### 3.1.1. General Versus Specific Parameters

Regarding the dependence between specificity and accuracy of the force field, it has been shown before that a more general and more transferable force field, such as UFF, using a smaller number of atom types, may perform, in many aspects, very well, or even better than one with more specific, fine-grained atom types [34]. With the exception of bond lengths, where fine-grained atom types are nearly always more accurate, other parameters do not benefit from a fine-graining of atom types, unless extreme effort is invested. That is virtually never the case for the parameters of a ligand or other molecular moiety unknown for a common biomolecular force field, see e.g., [13,35,36]. These are obtained with very little effort compared to the effort needed to obtain the basic force field for the common moieties, such as standard amino acid residues and nucleic acid bases. Together with low-level DFT or semiempirical calculations to derive the bonding and non-bonding (mainly electrostatic) parameters, the parameters of the unknown ligand, as conducted using the automated tools mentioned before, may not be superior to the UFF parametrization. The potential for higher accuracy in the case of finer-grained atom types may be misleading, resulting in no gain in liquid-state properties when parametrizing against even good-level DFT data. The reason is the simplicity of the force-field form common to all of, so-called, classical force fields [37] or force fields for classical molecular dynamics [38].

#### 3.1.2. More Accurate Parameters for Structure Refinement?

As further discussed in ref. [39], a higher accuracy requires another force-field form, including explicit polarizability and multipole expansion of the charge density (see e.g., ref. [40]). For the common, simpler force fields, parametrizing against the forces obtained from quantum chemical calculations generally results in inferior bulk properties, such as the density of the liquid and diffusion constant, as shown in the case of water parameters. For the polarizable, more complicated force field, the effort to include simultaneously different local and global properties into account is lacking [41]. Consequently, just the force-field form, without accurate parametrization, does not guarantee a higher predictive/interpretative power than the classical one, as shown for NMR-derived data, e.g., in ref. [42]. Clearly, concerns about the accuracy of the force field are relevant only for advanced molecular modeling tasks. For tasks focused on deriving standard ensembles from dense and accurate experimental restraints, approximate force field parameters are sufficient for refinement using the combined potential of the force field and the restraints.

To conclude, the UFF parametrization can be seen as valid and, in the vast majority of cases tested so far, sufficiently accurate for refinement of the NMR-derived structures of biomolecules and their complexes with ligands. For the two exceptions encountered so far, resulting in inaccurate bond lengths, ad hoc modifications have been introduced, as described in Section 4.

### 3.2. Strengths and Temporary Weaknesses of the Molecular Restrainer

We have seen that the molecular restrainer can refine structures independently of their complexity with very little user effort and computer time. These are the major strengths of the molecular restrainer. The molecular restrainer’s minimization also improves the already deposited PDB structures in some cases. Only two corrections of bond lengths were so far needed in cases of situations involving partial double bonds, as described in Materials and Methods. The current implementation, supporting distance restraints in the form of a CYANA .upl file, is useful, due to the ability of CYANA to deal with any molecular moiety with ease. Nevertheless, it is currently a restriction for those having the distance restraints ready in another format for/from other tools. A future update will also support the NMR exchange format (.nef) file and different restraint types, resolving this limitation. The current implementation of pseudoatoms for chemical groups with undistinguished assignment is not fully sufficient due to a lack of standardization in their naming convention. As an unfavorable example, for the particular pseudoatom named Q25 by CYANA, defined for the ABX ligands in this work, the number 25 has no relation to the hydrogen names or numbers that it represents, in which case SAMSON cannot use them. This will also be solved by using the .nef format that includes the full definitions of pseudoatoms. The vast majority of restraints are nevertheless handled correctly, making the current implementation already very useful. It is important to note that no single set of structures can perfectly represent the dynamic conformational ensemble from which NOE signals originate. The structures derived by conventional NMR spectroscopy are rather the starting points for further analysis or for further simulation studies. In that, the starting structure should be accurate, a goal to which this molecular restrainer clearly contributes. It should be chemically correct, for which SAMSON fixes the protein termini, and it should be unbiased, to which the restrainer contributes by releasing the rigid geometries of the amide bond planes and other restrictions imposed by the dihedral angle dynamics of CYANA.

## 4. Materials and Methods

### 4.1. Treatment of the Structure File, NMR-Specific Data

The use of the molecular restrainer starts with opening the set of NMR structures from CYANA in SAMSON (Supplementary Materials). These are normally missing the N-terminal amine protons and C-terminal “OXT’’ oxygen. In order to correct these, the molecular restrainer implements an option that fixes terminal residues based on their geometry. Only termini with standard amino acid residues would be fixed. Pseudoatoms are not expected in the loaded structure but are automatically created from the hydrogen atoms of the PDB structures, according to the following naming rules: pseudoatom Q is constructed from these parents: H1, H2, and H3; pseudoatom QX from HX1, HX2, and HX3; and pseudoatom QQX from HX11, HX12, HX13, HX21, HX22, and HX23, where X is an alphabetical character. Only a subset of these parent atoms may be present, such as HX2 and HX3 for a CH_2_ group. As an example, QA would be formed from HA1, HA2, and HA3 if these are present. These rules correctly create most pseudoatoms of the standard residues.

### 4.2. Distance Restraints, Force Field, and Structure Minimization

**Figure 3:**
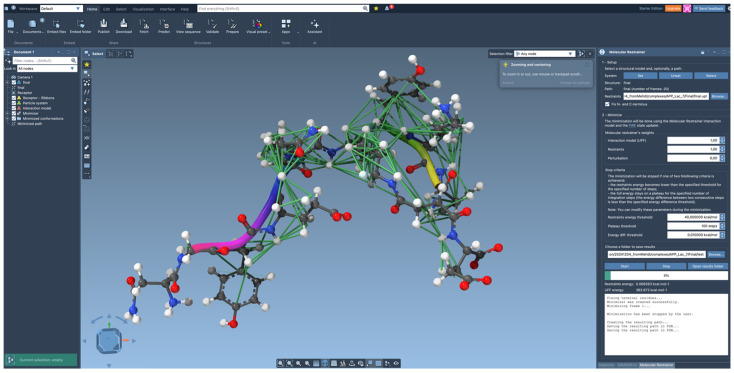
The molecular restrainer interface, integrated in SAMSON, with visualized distance restraints. The settings of the molecular restrainer, along with its informative progress report, are shown on the right. The green color of the visualized restraints indicates that distances are within the specified restraint limits.

NMR-derived restraints are loaded in the format used by CYANA (see Figure 3). Only the upper distance restraints are currently supported, obtained from the .upl file. Distance restraint entries that cannot be identified are ignored in the calculation and reported in the restraints.log file. By default, the distance restraints in the .upl file, including pseudoatoms, have pseudoatom corrections (*r*_p_) [5]. These are back-converted by SAMSON to actual distances r by knowing the number of protons (N_Q_) forming the pseudoatom Q using *r* = *r*_p_* *N*_Q_^(1/6)^. The pseudoatoms formed from hydrogen atoms at different parent atoms have a further correction for the interatomic distance. The force acting on the pseudoatom is evenly distributed to the parent atoms, and the “position” of the pseudoatom is the average of the positions of the corresponding hydrogen atoms. The restraining force follows a harmonic potential up to 5 Å from the set value, beyond which the force is kept constant. The default force constant *K* is 198.6 kcal mol^−1^ Å^−1^, but this can be scaled by the user. Basic atom interactions are treated by the UFF, currently with two modifications: the C-O distances in carboxylate COO^−^ were set to 1.25 Å and the CZ-N* bonds in arginine to 1.34 Å. These bonds are challenging for the atom-centered force field due to the partial double-bonded character.

For minimization using UFF together with distance restraints, the fast inertial relaxation engine (FIRE minimizer) [43] is implemented. FIRE is particularly effective for optimizing atomistic models with applied restraints due to its ability to efficiently navigate complex energy landscapes. By integrating inertial effects, FIRE accelerates convergence, making it significantly faster than traditional methods like steepest descent, especially in scenarios involving large-scale motions that minimally affect potential energy. This efficiency is crucial when restraints are present, as they can introduce additional energy barriers and local minima to those already present in UFF.

### 4.3. Algorithm for Structure Refinement

The setup of the forces for minimization in the combined potential of the UFF and the restraints is as follows. First, all forces resulting from each contribution of the force field are added, including the bond, angle, dihedral and van der Waals forces, calculated from all adjacent atom pairs, sets of three atoms forming angles, sets of four atoms forming torsion angles, and all (not just adjacent) atom pairs forming the nonbonding contacts for the van der Waals interactions. These are evaluated as vectors in Cartesian space, as in common molecular mechanics simulation code.

The restraints are added as bonding forces between pairs of (pseudo)atoms with a special form of ***F***_r_ = *K*∙*d*∙***n***, where ***n* =** (***p***_1_ − ***p***_2_)/|***p***_1_ − ***p***_2_| is the directional vector and *K* is the force constant and where *d* = min(|***p***_1_ − ***p***_2_|, *r*), with *r* being the upper distance set to 5 Å. This results in a constant force beyond the upper distance, commonly used for distance restraints [44].

### 4.4. Further Information

The “Molecular Restrainer” extension is tightly integrated into SAMSON and uses other SAMSON extensions internally to perform minimization, namely the universal force field [45] and the FIRE state updater [46]. The results obtained from the “Molecular Restrainer” are automatically exported into .pdb format and can be exported in additional formats for use in other software. The “Molecular Restrainer” is available in the SAMSON free plan.

## 5. Conclusions

We have demonstrated the performance of the molecular restrainer, a new tool within SAMSON—the platform for integrative molecular design—developed to refine NMR-derived structures. Its capability to handle molecules of a diverse chemical nature, combined with its ease of use, minimal user intervention, and computational cost, as well as its seamless integration into a modern molecular design environment, make it well-suited for widespread adoption.

## Figures and Tables

**Table 1:** Ligands or non-standard residues present in the studied PDB structures.

PDB Code of the Complex	PDB Code of the Ligand	Systematic Name *
2N3Y	1PA	4-(carboxymethyl)-L-phenylalanine
	MH0	mesoheme
2NBN	PLM	palmitic acid (COOH form!)
5LQV	PGM	1-myristoyl-2-hydroxy-sn-glycero-3-[phospho-rac-(1-glycerol)]
6R1V	JPT	6-(hydroxymethyl)-3-oxidanyl-2-(thiophen-3-ylmethyl)pyran-4-one
7NM2	UJ5	2-[(~[S])-methoxy-(4-propan-2-ylphenyl)methyl]-3~[H]-benzimidazole-5-carboxylic acid (COOH form!)
PDB name of the peptide	Non-standard chem. moiety	Further/alternative specification
APP-Lac-7	Lactam	Cyclic amide
APP-RCM-21	Aliphatic double bond	Forming a long aliphatic section of a cycle
APP-WT-4	N/A	N/A
APP-TRIAZ-31	Triazole ring	Five-membered ring as a part of a larger cycle
APP-X-lin-57	Organic sulfide	Thioether-containing cycle

* when available.

**Table 2:** Scores of the structures before and after minimization in SAMSON obtained from wwpdb validation server (first three pairs of columns) and from CYANA (last column pair). Lower values indicate better structural quality.

	Clashscore *		Ramachandran		Side Chain		CYANA TF	
2N3Y	0	1	1.3	2.2	7.0	5.3	4.18	0.93
2NBN	19	2	0.1	1.9	30.3	4.9	4.93	2.00
5LQV	14	4	0.2	2.0	10.2	3.5	160.57	22.23
6R1V	7	1	1.4	3.0	9.3	7.7	16.34	2.42
7NM2	10	2	1.2	0.4	18.3	7.9	3.74	1.82
APP-Lac-7	0	12 **	0.0	0.0	2.1	1.4	0.05	0.07
APP-RCM-21	0	0	0.0	0.0	3.6	0.7	0.19	0.46
APP-TRIAZ-31	0	0	0.0	0.0	20.0	20.0	0.15	0.77
APP-WT-4	0	1	0.0	0.0	11.4	3.3	0.03	0.04
APP-X-lin-57	0	0	0.0	0.0	15.0	14.3	0.22	0.30
abx-ABX	18	5	0.8	1.5	17.3	9.8	7.09	9.38

* The two columns represent the values before and after minimization in SAMSON, left, right, respectively. ** The validation server fails to correctly identify bonds in non-standard residues; the identified clashes are therefore due to internal errors of the validation server.

## Data Availability

SAMSON is available according to ref. [18] with Molecular Restrainer available in the free plan.

## Supplementary Materials

The following supporting information can be downloaded at: https://www.mdpi.com/article/10.3390/ijms26115091/s1.
