# Supplementary material for: Streamlined Postprocessing of NMR Structures with the Molecular Restrainer: A Universal Tool for High-Quality Protein–Ligand Models and Non-Standard Amino Acid Residues
Source: Int J Mol Sci. 2025 May 26;26(11):5091. doi: 10.3390/ijms26115091 (PMC12155397; doi:10.3390/ijms26115091)
Supplement: Supplementary file 1 [file ijms-26-05091-s001.zip › ijms-3576460-supplementary.pdf]

International Journal of *Molecular Sciences*

MDPI

Article

Supplementary information for: Streamlined Postprocessing of NMR Structures with The Molecular Restrainer: A Universal Tool for High-Quality Protein-Ligand Models and Non-Standard Amino Acid Residues

Jiří Mareš [1], Guneet Singh Tarang [1], Dmitriy Marin [2], Mehdi Mobli [3], Stephane Redon [2] and Julien Orts [1,*]

[1] Department of Pharmaceutical Sciences, University of Vienna, Josef-Holaubek-Platz 2, 1090 Vienna, Austria; jiri.mares@univie.ac.at (J.M.); guneet.singh.tarang@univie.ac.at (G.S.T.)
[2] OneAngstrom, 38000 Grenoble, France; dmitriy.marin@oneangstrom.com (D.M.); stephane.redon@oneangstrom.com (S.R.)
[3] Australian Institute for Bioengineering and Nanotechnology, University of Queensland, St Lucia, QLD 4072, Australia; m.mobli@uq.edu.au
* Correspondence: julien.orts@univie.ac.at

1. Installation

1.1 Create an account on SAMSON Connect

1.2 Download and install SAMSON

1.3 Add the "Molecular Restrainer" extension from SAMSON Connect Marketplace

1.4 Restart SAMSON - the extension will be automatically installed.

1.5 You can find it in SAMSON in Home > Apps > All > Molecular Restrainer.

2. Instructions for the user

After installing the “Molecular restrainer” and FIRE apps for SAMSON, the whole NMR structures ensemble is minimized in these few steps:

1. Load the file with your molecular system in SAMSON (e.g. a DPB file)
2. Open "Molecular Restrainer" in SAMSON and perform in it the following steps:

2.a. Click "Set" to set the system. If you have multiple systems loaded, you will need to first select the system from the document.

2.b Choose the file with the NMR restraints - a UPL file.

2.c Specify the Molecular Restrainer options, e.g. whether to fix termini, restrainer weights and stopping criteria.

2.d Choose the results folder.

2.e Click "Start" - it will automatically perform minimization with the given restraints and output the log and resulting files in the chosen folder.
